# Minocycline Inhibits Alkali Burn-Induced Corneal Neovascularization in Mice

**DOI:** 10.1371/journal.pone.0041858

**Published:** 2012-07-25

**Authors:** Ou Xiao, Zhao-lian Xie, Bin-wu Lin, Xiao-fang Yin, Rong-biao Pi, Shi-you Zhou

**Affiliations:** 1 The State Key Laboratory of Ophthalmology, Zhongshan Ophthalmic Center of Sun Yat-sen University, Guangzhou, China; 2 Department of Pharmacology & Toxicology, School of Pharmaceutical Sciences, Sun Yat-Sen University, Guangzhou, China; Wayne State University, United States of America

## Abstract

The purpose of this study was to investigate the effects of minocycline on alkali burn-induced corneal neovascularization (CNV). A total of 105 mice treated with alkali burns were randomly divided into three groups to receive intraperitoneal injections of either phosphate buffered saline (PBS) or minocycline twice a day (60 mg/kg or 30 mg/kg) for 14 consecutive days. The area of CNV and corneal epithelial defects was measured on day 4, 7, 10, and14 after alkali burns. On day 14, a histopathological examination was performed to assess morphological change and the infiltration of polymorphonuclear neutrophils (PMNs). The mRNA expression levels of vascular endothelial growth factor (VEGF) and its receptors (VEGFRs), basic fibroblast growth factor (bFGF), matrix metalloproteinases (MMPs), interleukin-1α, 1β, 6 (IL-1α, IL-1β, IL-6) were analyzed using real-time quantitative polymerase chain reaction. The expression of MMP-2 and MMP-9 proteins was determined by gelatin zymography. In addition, enzyme-linked immunosorbent assay was used to analyze the protein levels of VEGFR1, VEGFR2, IL-1β and IL-6. Minocycline at a dose of 60 mg/kg or 30 mg/kg significantly enhanced the recovery of the corneal epithelial defects more than PBS did. There were significant decreases of corneal neovascularization in the group of high-dosage minocycline compared with the control group at all checkpoints. On day 14, the infiltrated PMNs was reduced, and the mRNA expression of VEGFR1, VEGFR2, bFGF, IL-1β, IL-6, MMP-2, MMP-9, -13 as well as the protein expression of VEGFR2, MMP-2, -9, IL-1β, IL-6 in the corneas were down-regulated with the use of 60 mg/kg minocycline twice a day. Our results showed that the intraperitoneal injection of minocycline (60 mg/kg b.i.d.) can significantly inhibit alkali burn-induced corneal neovascularization in mice, possibly by accelerating corneal wound healing and by reducing the production of angiogenic factors, inflammatory cytokines and MMPs.

## Introduction

Corneal neovascularization (CNV) is a sight-threatening condition usually associated with inflammatory or infectious disorders of the ocular surface. Corneal chemical burn usually causes abundant CNV in which severe inflammation and corneal lysis are involved [1].Nowadays, most work searching for antiangiogenic agents is focused on antiangiogenic factors [2], [3], anti-inflammation [4], [5], or anti-remodeling of extracellular matrix [6], [7], separately.

In recent years, tetracyclines have been demonstrated to have the potential of inhibiting all of the above processes during pathological neovascularization, due to its newly discovered non-antibiotic properties,includinganti-inflammation [8],antiangiogenesis [9],anti-apoptotic [10], inhibition of the production and activity of matrix metalloproteases [11], etc.

Minocycline and doxycycline are two widely used second-generation, semi-synthetic tetracycline analog. Doxycycline has shown a capacity for inhibiting CNV in rats by topical and systemic use [7], [9], [12]. Minocycline has seldom been used in CNV models. It was just demonstrated successful in inhibiting tumor-induced rabbit CNV [13]. Although both doxycycline and minocycline have similar effects on inhibiting human aortic smooth muscle cell migration, Yao's experiments showed that minocycline seemed to have more versatile effects because it not only inhibited MMPs but also down-regulated ERK1/2 and Akt pathways [14].Furthermore, minocycline can readily cross the blood–brain barrier with a rate of at least fivefold higher than doxycycline in rodents [15]. These advantages of minocycline may explain why it has been widely used in clinical practice as a non-antibiotic agent for neurodegenerative diseases, such as multiple sclerosis [16], spinal cord injury [17], amyotrophic lateral sclerosis [18], Huntington's disease [19], and Parkinson's disease [20]. Most of the effects exerted by minocycline are related to its inhibitory activity on inflammation, matrix metalloproteases, and/or apoptotic cell death [21]. In order to determine whether these multi-targets of minocycline will function in alkali burned-induced CNV, we treated mice with intraperitoneal minocycline in this study.

## Materials and Methods

### Animals

A total of 105 female BALB/c mice, aged 4–5 weeks, and weighing 15–19 g were purchased from Guangdong Provincial Center for Animal Research, Guangzhou, China. The right eye of each mouse was selected for the experiment. All experimentation on animals was conducted in accordance with the ARVO Statement for the Use of Animals in Ophthalmic and Vision Research. In addition, the research protocol was approved by the Animal Care Committee of the Zhongshan Ophthalmic Center at Sun Yat-sen University (approval ID: 2008-009, Guangzhou, China).

### Induction of corneal neovascularization (CNV)

Corneal neovascularization was induced by alkali injury using a previously described method with little modification [22].In brief, after general anesthesia with an intraperitoneal injection of 4.6% chloral hydrate (10 ml/kg) and topical anesthesia with a drop of 0.5% proparacaine hydrochloride (Alcaine eye drops, Alcon Inc., Fort Worth, TX, USA), a filter paper of 2.5 mm in diameter soaked with 2.5 µl NaOH 0.1 M was placed on the central cornea for 40 s, followed by immediate rinsing with 30 ml of 0.9% saline solution for 10 s. The entire corneal limbus and epithelium were then scraped off with a surgical blade under a microscope. Tobramycin ophthalmic ointment (Tobrex, Alcon Inc.) was administered after the operation.

### Animal treatment

The mice were randomly assigned to three groups (35 mice per group) and treated with intraperitoneal injections of either PBS or minocycline (Sigma-Aldrich, St. Louis, MO, USA) twice a day for 14 consecutive days. The control group was treated with PBS (10 ml/kg) with every injection. The high-dosage group was treated with 60 mg/kg minocycline (6 mg/ml), and the low-dosage group with 30 mg/kg minocycline (3 mg/ml).

In animal research on neurodegenerative diseases, minocycline was mostly used by intraperitoneal injection; its single dosage ranged from 5 mg/kg to 90 mg/kg [23]. The maximal serum concentration of minocycline is attained within 1–4 hours after oral administration, and the average half-life is around 15 hours [23]. So we decide to use both 30 mg/kg and 60 mg/kg every 12 hours in this study.

### Quantification of corneal neovascularization and measurement of corneal epithelial defects

On the 4th, 7th, 10th, and 14th day after alkali burns, the mouse corneas were examined and photographed with a digital camera (Cannon, Tokyo, Japan) attached to a slit-lamp microscope(SL-120; Zeiss Inc., Jena, Germany). Corneal epithelial defects were shown by 0.5% fluorescein staining of the ocular surface and by observation under cobalt blue light. Three consecutive photos with satisfactory full-face imaging were used for image analysis. Image J software(ver. 1.62) was downloaded from the website of U.S. National Institutes of Health (NIH) and used for quantitative analysis, as it was in a previous report [24].The areas of CNV and epithelial defects were measured and their percentages to the entire cornea were calculated.

### Histopathological and immunohistochemistrical examination

Fourteen days after alkali burns, 5 mice in each group were randomly sacrificed and their eyeballs were enucleated for histopathological and immunohistochemistrical examination. The eyeballs were fixed in a 10% neutral buffered formaldehyde solution and embedded in paraffin. The paraffin blocks of samples were cut until the first slide with corneal tissue was seen. Then the next three 20th slides of 5 µm thick were collected for immunostaining, and another three 25th slides for hematoxylin-eosin staining. For immunostaining of PMNs, these sections were deparaffinized and boiled in antigen retrieval solution (DAKO, Glostrup, Denmark) for 15 min. Then endogenous peroxidase activity was blocked by 3% hydrogen peroxide for 15 min. Nonspecific staining was blocked by 1% BSA for 1 hour. Sections were then incubated with the primary antibody (monoclonal rat antibody against mouse neutrophil marker NIMP-R14; sc59338, Santa Cruz Biotechnology, Santa Cruz, CA) diluted in 1% BSA (1∶300) overnight at 4°C. After three washes with PBS for 15 min, they were incubated with HRP-conjugated secondary anti-rat IgG antibody (ZSGB-BIO Institute of Biotechnology, Beijing, China) for 1 hour at room temperature and washed again with PBS. The reaction product was developed with 3, 3N-diaminobenzidine tertrahydrochloride (DAB) for 15 s. Then the sections were counterstained with Mayer hematoxylin. For histopahtological study, the corneal sections were procured, as in the procedures for immunostaining, and the slides were deparaffinized and regularly stained with hematoxylin-eosin. Corneal morphology and images of the infiltrated PMN were assessed by light microscopy. The number of infiltrated inflammatory cells and PMNs in the corneal stroma was counted in five randomly selected fields (×400) of a slide.

### Analysis of mRNA expression

Real-time quantitative reverse transcription polymerase chain reaction (real-time qRT-PCR) was performed to detect the mRNA expression levels of gene VEGF, VEGFR1 and 2, bFGF, TNF-α, IL-1α, IL-1β, IL-6, and MMP-2, -8, -9, -13 in the murine corneas. On the14th day after the burns, 7 mice of each group were euthanized by cervical dislocation, and their right corneas were procured. Total RNAs of each cornea were immediately isolated using an RNeasy kit (Qiagen, Valencia, CA, USA). After quantification of the RNA concentration, total RNA was treated with DNase I (Sigma-Aldrich) to remove any contaminated genomic DNA. A total of 0.5 µg RNA was reversely transcribed into cDNA in a 20-µl volume reaction system, using the Maxima® First Strand cDNA Synthesis Kit (Fermentas International Inc., Burlington, ON, Canada). Samples of synthesized cDNA were divided into aliquots and stored at −80°C.

Real-time qRT-PCR was performed and analyzed using an ABI PRISM 7000 sequence detection system (Applied Biosystems Inc., Foster City, CA, USA). PCR was performed in a 20-µl volume reaction system containing 10 µl 2×SYBR Green reaction mix (Invitrogen, Carlsbad, CA, USA), 0.4 mmol/L paired primers, and 1 µl cDNA. The sequences of the PCR primer pairs are listed in Table 1. Thermal cycling consisted of denaturation for 3 min at 95°C, followed by 40 cycles of 15 s at 95°C and 30 s at 60°C. The PCR amplification efficiency of the primer sets has been determined to be essentially 100% before qPCR. A comparative Ct (ΔΔCT) method was used to compare the mRNA expression levels of genes of interest. The gene of GAPDH was chosen as an internal control gene.

**Table 1:** Primer sets for real-time PCR.

Gene	Forward (5′-3′)	Reverse (5′-3′)
**GAPDH**	CACATTGGGGGTAGGAACAC	CTCATGACCACAGTCCATGC
**VEGF**	TTACTGCTGTACCTCCACC	ACAGGACGGCTTGAAGATG
**VEGFR1**	GTGATCAGCTCCAGGTTTGACTT	GAGGAGGATGAGGGTGTCTATAGGT
**VEGFR2**	CTGTGAACGCTTGCCTTAT	CAACATCTTGACGGCTACTG
**bFGF**	CCAACCGGTACCTTGCTATGA	TTCGTTTCAGTGCCACATACCA
**TNF-α**	CAGCCTCTTCTCATTCCTGCTTG	GGGTCTGGGCCATAGAACTGA
**IL-1α**	CTCTAGAGCACCATGCTACAGAC	TGGAATCCAGGGGAAACACTG
**IL-1β**	CTCCATGAGCTTTGTACAAGG	TGCTGATGTACCAGTTGGGG
**IL-6**	CAAAGCCAGAGTCCTTCAGA	GATGGTCTTGGTCCTTAGCC
**MMP-2**	CCCCGATGCTGATACTGA	CTGTCCGCCAAATAAACC
**MMP-8**	GATTATGGAAATGCCTCG	CTTCAGCCCTTGACAGC
**MMP-9**	CAGCCAACTATGACCAGGAT	CTGCCACCAGGAACAGG
**MMP-13**	GTGTGGAGTTATGATGATGT	TGCGATTACTCCAGATACTG

### Gelatin zymography

The relative amount of MMP-2 and MMP-9 proteins in the corneas was measured by gelatin zymography, in accordance with a previous report with little modification [25]. In brief, corneas in each group (n = 5, every four of 20 corneas in each group were randomly divided and pooled into five samples) were homogenized at 4°C in 50 mM Tris–HCl, pH 7.4, which included a protease inhibitor cocktail that did not contain EDTA (Fermentas International). Supernatants from each tissue homogenate were collected after centrifugation at 15,000 rpm for 10 min at 4°C. Total proteins in the supernatant were quantified with a commercial kit (BCA Protein Assay Kit, Beyotime Institute of Biotechnology, Shanghai, China). Protein of 15 µg from each sample was mixed with 5X non-reducing sample buffer (4∶1, v/v) containing sodium dodecyl sulfate (SDS). Samples were loaded onto 7.5% SDS polyacrylamide gels containing 0.1% porcine skin type A gelatin (Sigma-Aldrich) as a substrate for MMP-2 and MMP-9 functions. After electrophoresis, the gels were rinsed in 2.5% Triton X-100 (Sigma-Aldrich) to remove SDS and incubated in 0.05 M Tris-HCl buffer containing 5 mM CaCl2 for 16 hours at 37°C. After incubation, the gels were stained with 0.2% Coomassie blue (Sigma-Aldrich) and destained in a methanol/acetic (40%/10%) acid solution until bands of gel digestion were visible. The gels were imaged with a G:BOX BioImaging system (Syngene, Cambridge, England), and the pictures were analyzed with Image J (ver. 1.62) software to obtain densitometry readings of digested gelatin bands for semi-quantitative analysis.

### Enzyme-linked immunosorbent assay (ELISA)

The supernatants obtained from the above protein extraction procedures were used to determine the protein levels of VEGFR1, VEGFR2, IL-1β and IL-6 with commercial ELISA kits (IL-1β, Invitrogen; VEGFR1 and 2, IL-6, R&D Systems Inc., Minneapolis, MN, USA) according to the manufacturers' instructions. The data were expressed as the target molecule (picograms) per total protein (milligrams) for each sample.

### Statistical analysis

Statistical analysis was performed using the SPSS (vers. 16.0) software for Microsoft Windows XP (SPSS Inc., Chicago, IL, USA). All data are shown as mean ± standard deviation. Data were compared using one-way ANOVA among the three groups, and Least-significant difference (LSD) analysis was performed to compare every two groups. A *p* value of less than 0.05 was considered statistically significant.

## Results

### Effects of minocycline on alkali burn-induced CNV

Two mice in the control group and three in each of the two treatment groups died from anesthesia during the examination on day 10 after the chemical burns.

**Figure 1:**
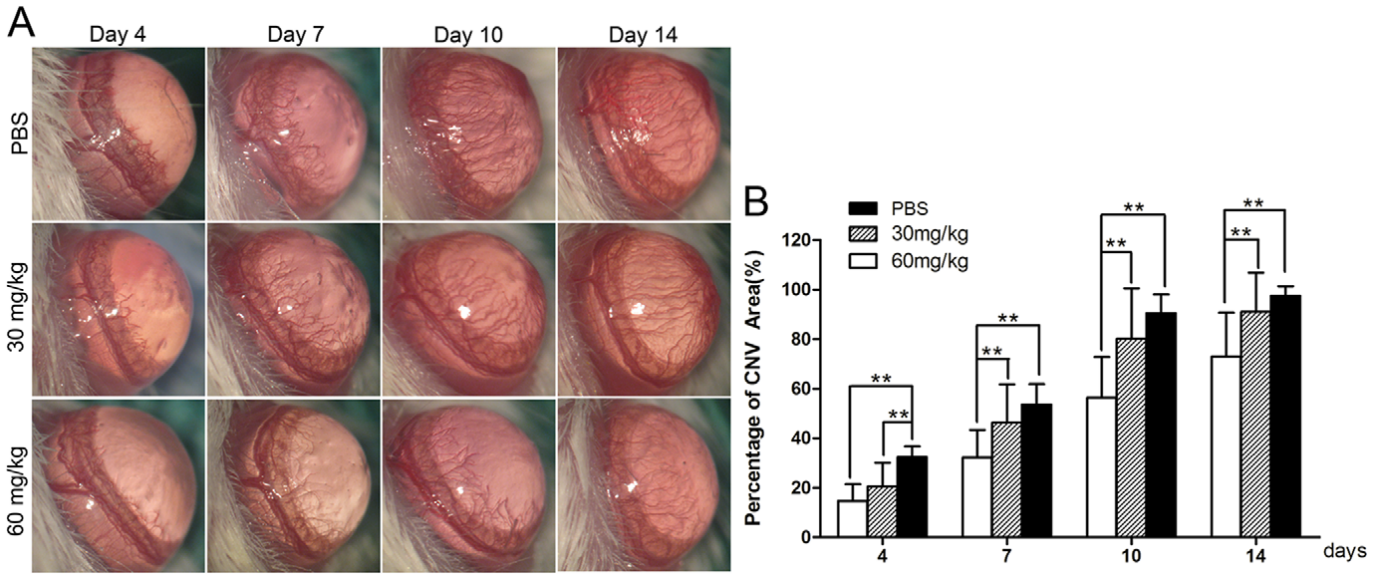
Inhibitory effect of minocycline on corneal neovascularization (CNV). (**A**) Representative images of CNV on day 4,7,10 and 14 after alkali burns treated with intraperitoneal injection of PBS, minocycline (30 mg/kg b.i.d) or minocycline (60 mg/kg b.i.d). (**B**) The percentages of CNV area in the three groups at different checkpoints. The percentage of CNV area in the high-dosage group reduced significantly compared with the control group. There was no statistical difference in the percentage of CNV area between the low-dosage group and control group after injury, except on the fourth day. (n = 32, **p<0.01).

The area of CNV increased over time in all three groups, while shorter and fewer new corneal vessels were found in the high-dosage group than in the other groups at every checkpoint (Fig. 1A). There were significant differences in the percentages of CNV area among the three groups at every checkpoint after alkali injury (all *p*<0.01; Fig. 1B). The percentage of CNV area in the high-dosage group reduced significantly when compared with the control group (14.61%±6.84% versus 32.39%±4.35% on day 4, 32.19%±11.15% versus 53.68%±8.19% on day 7, 56.43%±16.42% versus 90.53%±7.46% on day 10, and 73.03%±17.81% versus 97.43%±3.91% on day 14, all *p*<0.01; Fig. 1B). Otherwise, there was no statistical difference in the percentage of CNV area between the low-dosage group and control group after injury, except on the 4th day (20.62%±9.47% and 32.39%±4.35%, *p*<0.01; Fig. 1B).

### Minocycline promoted corneal epithelial recovery

**Figure 2:**
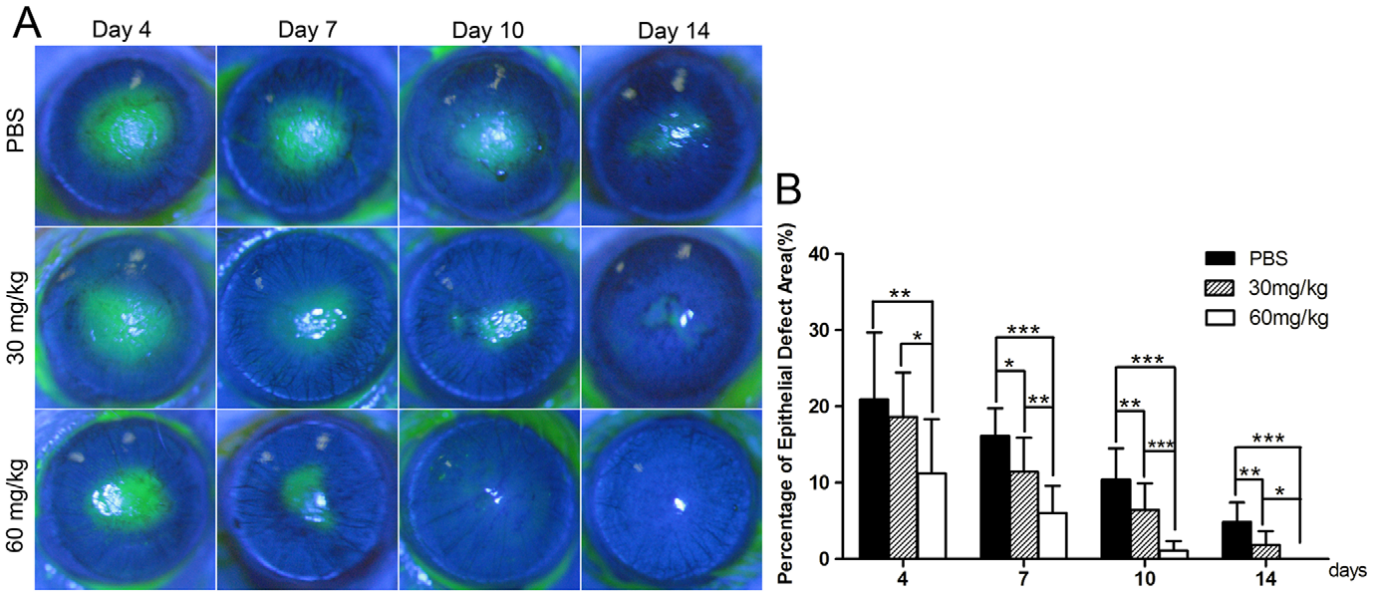
Minocycline promoted corneal epithelial recovery after alkali burns. (**A**) Representative images of the mice corneas with fluorescein staining with intraperitoneal injection of minocycline or PBS after alkali burns. (**B**) The area of epithelial defect in the minocycline-treated groups was significantly smaller than that in the control group. The percentages of corneal epithelial defect in the control group, the low-dosage group, and the high-dosage group were 20.92%±8.77%, 18.62%±5.83% and 11.19%±7.14% on day 4; 16.13%±3.63%, 11.41%±4.47% and 6.02%±3.56% on day 7; 10.40%±4.09%, 6.42%±3.50% and 1.11%±1.24% on day 10; 4.84%±2.58%, 1.85%±1.80% and 0% on day 14, respectively. (*p<0.05,**p<0.01, ***p<0.001).

Corneal epithelial defect was shown by positive fluorescein staining. The results showed that the recovery of corneal epithelial defect was faster in the minocycline-treated groups. The cornea surface was positive staining in punctuate on day 10 in the high-dosage group and negative fluorescein staining on day 14. In the low-dosage group, positive fluorescein staining was also not obvious on day 14, while in the control group some small epithelial defects were still noted (Fig. 2A). There were significant differences in the improvement of epithelial defects among the three groups at every checkpoint after alkali burns (all *p*<0.05; Fig. 2B). The percentage of epithelial defect area in the high-dosage group reduced significantly when compared against the control group (11.19%±7.14% versus 20.92%±8.77% on day 4, 6.02%±3.56% versus 16.13%±3.63% on day 7, 1.11%±1.24% versus 10.40%±4.09% on day 10, 0% versus 4.84%±2.58% on day 14, all *p*<0.01; Fig. 2B). The differences also existed between the low-dosage group and the control group 7 days after injury (11.41%±4.47% versus 16.13%±3.63% on day 7,6.42%±3.50% versus 10.40%±4.09% on day 10, 1.85%±1.80% versus 4.84%±2.58% on day 14, all *p*<0.05; Fig. 2B).

### Minocycline alleviated inflammation and reduced the infiltration of polymorphonuclear neutrophils (PMNs) in the alkali-burned mice corneas

**Figure 3:**
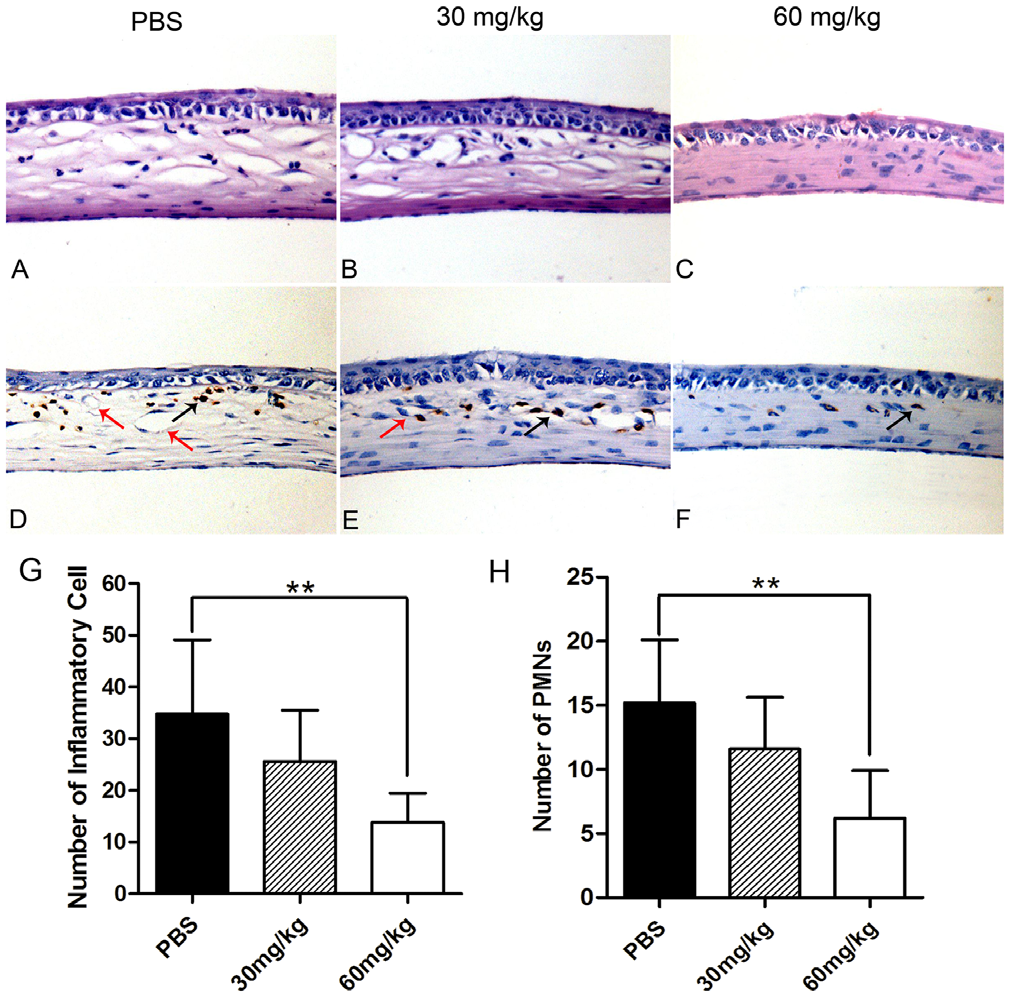
Anti-inflammatory effects of minocycline on the burned corneas at day 14. (**A–C**) H–E staining of the mice cornea. (Magnification, ×400) The corneal thickness was increased in the control group and the low-dosage group than that in the high-dosage group. The number of infiltrated inflammatory cells in one field was lower in the corneal stroma of the high-dosage group (13.8±5.7) than that in the control group (34.8±14.3) (**G**). (**D–F**) Immunohistochemistrical staining of PMNs. The PMNs were labeled in brown and the endothelium of corneal vessels was labeled with red arrows. The number of infiltrated PMNs (black arrows) in one field was lower in the corneal stroma of the high-dosage group (6.2±3.7) than that of the control group (15.2±5.0). There were no statistical differences of inflammatory cells or PMNs between low-dosage group and control group (**H**). (*p<0.05).

Histopathological analysis of the cornea revealed that there was a significant reduction in the number of infiltrated inflammatory cells (mainly as polymorphonuclear neutrophil, macrophage and lymphocyte) in the minocycline (60 mg/kg b.i.d) treated group when compared with the control group (*p* = 0.009; Fig. 3A–C,3G). In the control group, the corneal stroma was thicker and more swollen than that in the high-dosage group (Fig. 3A–C). The number of infiltrated PMNs in the corneal stroma was significantly lower in the high-dosage group than that in the control group (*p* = 0.006; Fig. 3H). There was no statistical difference in the infiltrated inflammatory cells or PMNs between the low-dosage group and the control group (Fig. 3G, 3H).

### Effects of minocycline on the mRNA expression of angiogenic genes in alkali-burned mice cornea

**Figure 4:**
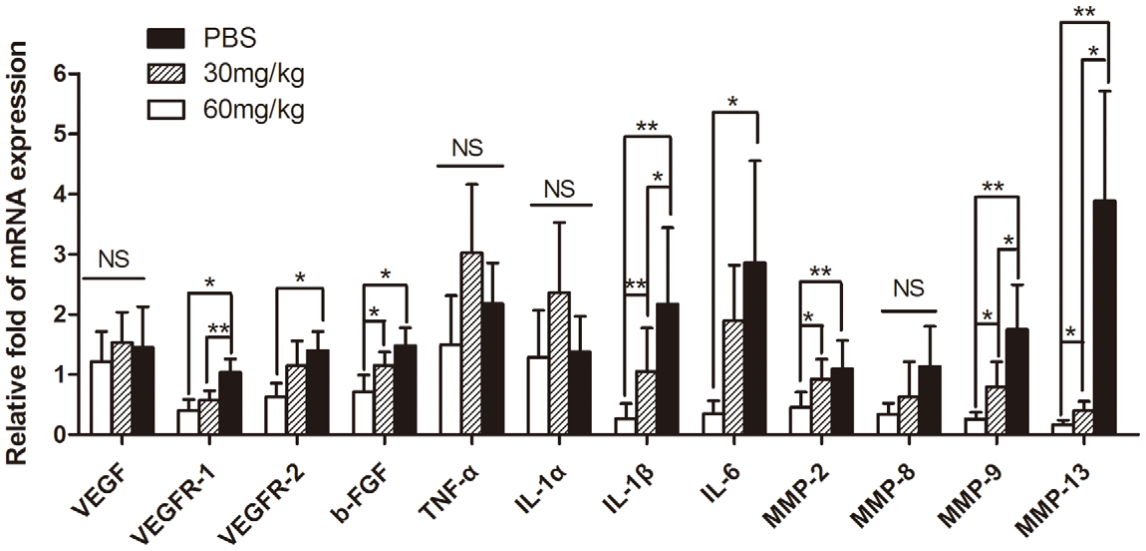
The mRNA expression of angiogenesis-related genes detected by real-time RT-PCR. All the data represented the relative fold change of mRNA expression of the genes of interest. Gene expression in the mice corneas 14 days after alkali burn was significantly lower in the high-dosage drug group: the expression levels were VEGFR1 38.46%, VEGFR2 45%, bFGF 47.97%, IL-1β 12%, IL-612%, MMP-2 41%, IL-1β 48%, MMP-9 14.94%, and MMP-13 4.37% of those in the control group.(n = 7, *p<0.05; **p<0.01).

The mRNA expression levels of VEGF and its receptors VEGFR 1 and 2, bFGF, TNF-α, IL-1α, IL-1β, IL-6, and MMP-2, -8, -9, -13 were examined and compared by quantitative real-time RT-PCR. The differences in the expression of VEGFR1 and 2, bFGF, IL-1β, IL-6, and MMP-2, -9, -13 among the three groups had statistical significance (all *p*<0.05; Fig. 4). The mRNA expression of these genes was significantly lower in the high-dosage group than that in the control group. The mRNA levels of VEGFR1, IL-1β, MMP-9, and MMP-13 were lower in the low-dosage group than that in the control group (all *p*<0.05; Fig. 4). However, there were no statistical differences among the three groups in the expression of genes VEGF, IL-1α, TNF-α, and MMP-8.

### Effects of minocycline on the production of angiogenesis-related proteins in the alkali-burned mice corneas

**Figure 5:**
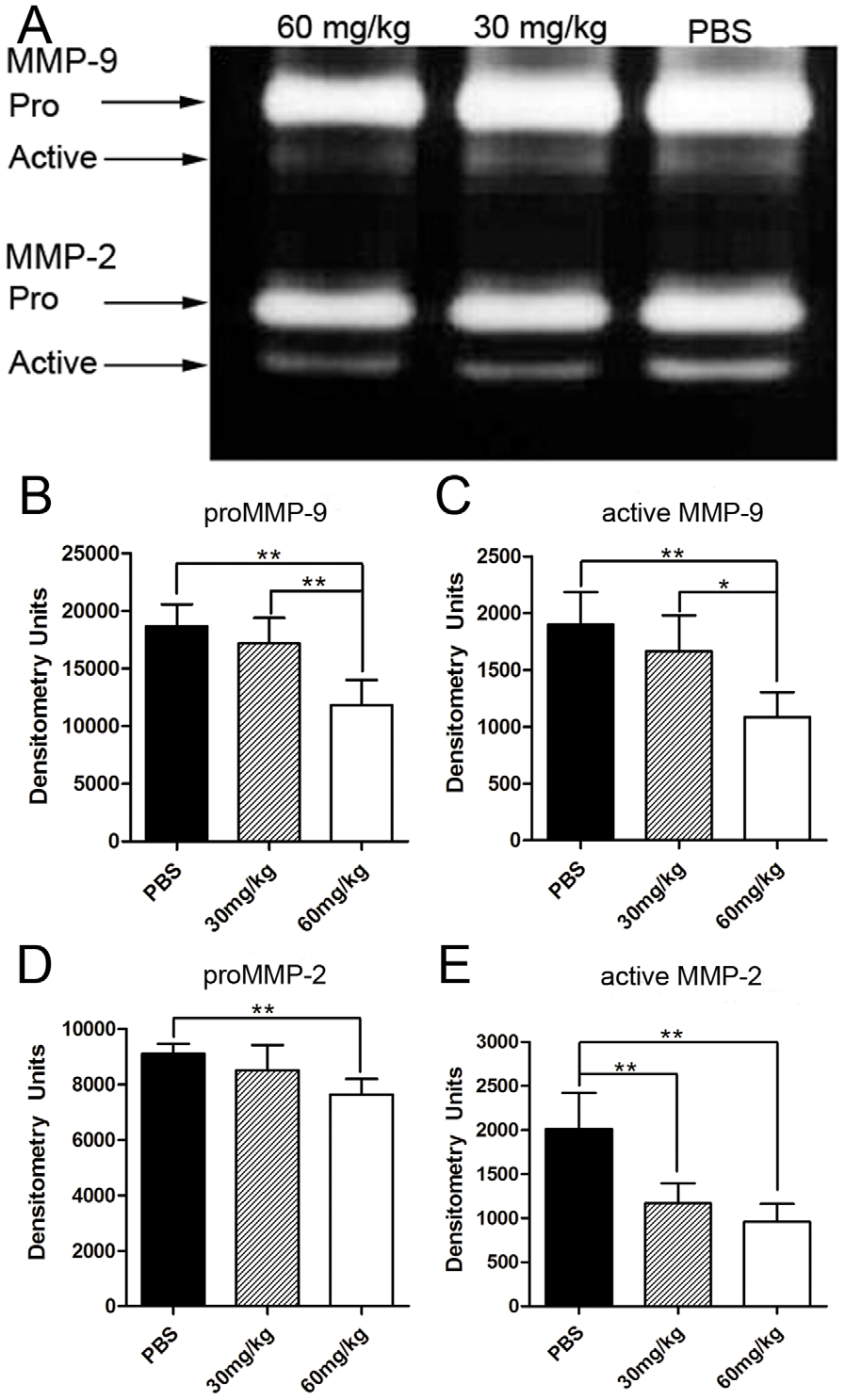
Gelatin zymography for MMP-2 and -9. (**A**) Representative image of gelatin zymography for MMP-2 and -9 of the corneas 14 days after alkali burns, treated with intraperitoneal injection of PBS, minocycline (30 mg/kg b.i.d) and minocycline (60 mg/kg b.i.d). (**B**) Densitometry analysis showed intraperitoneal injection of minocycline (60 mg/kg b.i.d) significantly reduced the protein expression of MMP-2 and MMP-9 proenzymes and their activated forms in mouse corneas than that in the low-dosage group and the control group (except for the activated MMP-2 protein). (n = 5,*p<0.05; **p<0.01).

**Figure 6:**
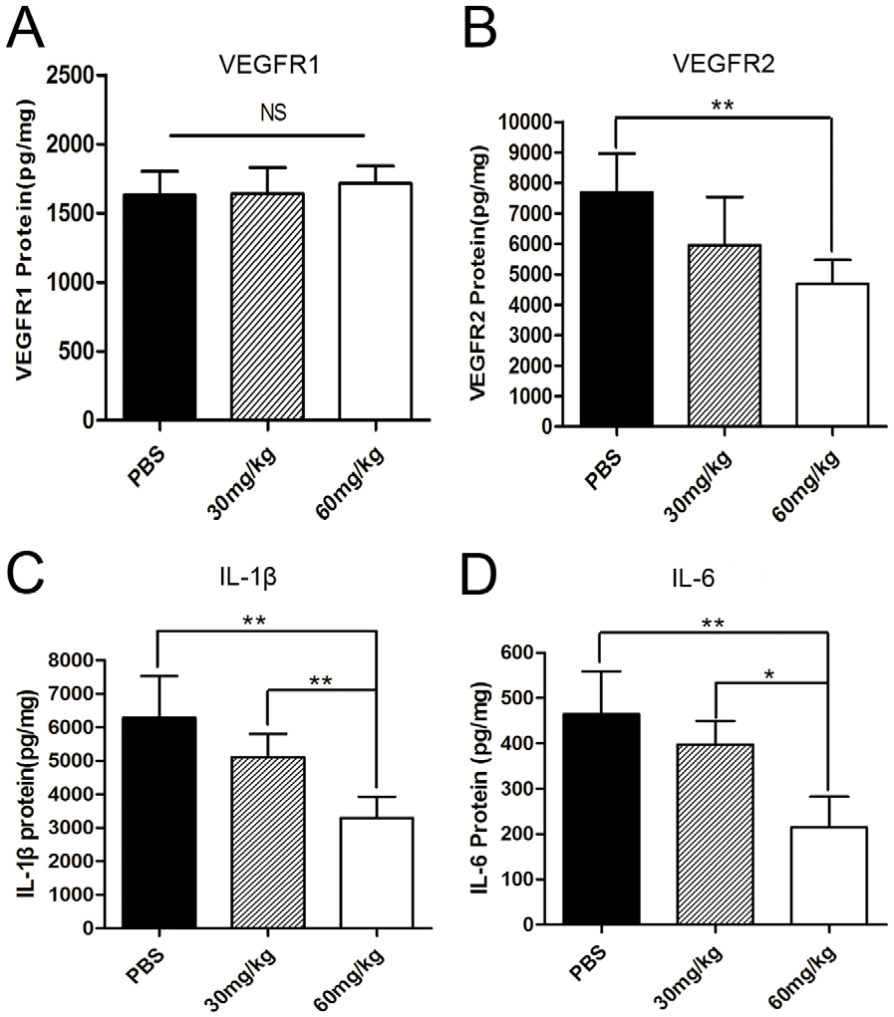
The expression of VEGFR1, VEGFR2, IL-1β and IL-6 proteins detected by ELISA. The expression of VEGFR2 protein in the mice corneas of the control group, the low-dosage group, and the high-dosage group were 7688.31±1274.27, 5956.76±1579.41 and 4691.27±776.67 pg/mg, IL-1β protein was 6285.41±1246.17, 5107.15±694.55 and 3295.47±631.90 pg/mg, and IL-6 protein was 464.06±94.55, 397.90±51.64, and 214.98±67.90 pg/mg, respectively. The protein levels of VEGFR2, IL-1β and IL-6 were lower in the high-dosage group than that in the control group. There were no statistical differences in the expression of VEGFR1 protein among the three groups. (n = 5, *p<0.05; **p<0.01).

To investigate the relative quantity of matrix metalloproteinases in the burned mouse corneas, the expression levels of MMP-2 and MMP-9 proteins were tested by gelatin zymography (Fig. 5). The protein levels of VEGFR1,VEGFR2,IL-1β and IL-6 were determined by ELISA (Fig. 6). The results showed that there were significant differences in the production of all the above proteins, except with VEGFR1 among the three groups (all *p*<0.05 Fig. 5B–E, Fig. 6B–D). Further analysis showed that the production of MMP-2 and 9 proenzymes and their activated forms, VEGFR2, IL-1β and IL-6 proteins were significantly reduced in the high-dosage group when compared with the control group (all *p*<0.01; Fig. 5B–E, Fig. 6B–D). There were no statistical differences in the expression of the above proteins except the activated MMP-2 between the low-dosage group and the control group (activated MMP-2, *p*<0.01; Fig. 5E).

## Discussion

Minocycline, a semi-synthetic tetracycline analog used in the treatment of many infectious diseases, was first described in 1967 [26]. In the recent years, many interesting properties of minocycline besides its antibiotic activity, such as anti-inflammation [21] and anti-apoptosis [10], have been identified. In this study, we found that intraperitoneal injection of minocycline at a dosage of 60 mg/kg/b.i.d could effectively inhibit CNV in mice after alkali injury. However, intraperitoneal injection of minocycline at a dosage of 30 mg/kg could only inhibit CNV at the early stage (day 4) and not keep its effective inhibition in the later follow-up. It seemed that the blood concentration of minocycline must reach a threshold to exert therapeutic effects continuously for inhibiting CNV.

The formation of CNV is a complicated pathological process consisting of two phases. One phase is the angiogenic growth factor (VEGF and bFGF) dependent on proliferation of vascular endothelium. The other is the remodeling of extracellular matrix components and the activation of cytokines. In order to further explore the mechanisms of minocycline in this study, we examined the expression of some main angiogenesis-related cytokines, including angiogenic factors, inflammatory cytokines, and MMPs. In this study, minocycline did not reduce the mRNA expression of VEGF; however, it did downregulate the mRNA and protein expression of its receptor 2. VEGFR2 was demonstrated to be the predominant mediator of VEGF-stimulated endothelial cell migration, proliferation, survival, and enhanced vascular permeability [27]. Otherwise, the expression of bFGF was inhibited by the use of minocycline in this study. Basic FGF is another major angiogenic factor and has a more potent effect on the induction of CNV than VEGF at the same dosage [28].

Alkali burn-induced CNV is closely related to inflammation. After chemical burns, the inflammatory cells will be recruited into the injured cornea and will release inflammatory cytokines and MMPs. They will then induce the formation of CNV and retard corneal epithelial recovery [1], [25]. Interleukin-1, -6, and TNF-α are important pro-inflammatory cytokines in the induction of CNV [29]. Some studies have shown that reducing the production of IL-1 or blocking its receptor IL-1R could inhibit CNV [4], [30], [31]. In our study, the mRNA expression levels of IL-1α and TNF-α did not show significant differences among the three groups 14 days after corneal burn, although minocycline did attenuate the development of diabetic neuropathic pain, partly by reducing the level of TNF-α [32].Our results showed that the expression of IL-1β mRNA and protein in the high-dosage group was significantly lower than that in the other two groups. This effect may be through inhibiting the production and activity of MMP-2 and MMP-9 by minocycline, as shown in this study. Previous studies have demonstrated that not only the IL-1β-converting enzyme (caspase-1) [33],but also MMP-2, -3, and -9 could activate the IL-1β precursor into mature IL-1β in inflammation [34]. Minocycline was also able to suppress the activation of caspase-1 [21]. Furthermore, in this study, the enhancement of corneal wound healing was noted by use of minocycline as in the previous reports of tetracycline and doxycycline [35], [36]. The improvement of corneal wound will allow less infiltration of inflammatory cells, and will then reduce the formation of corneal vessels. The above facts showed that minocycline could effectively inhibit angiogenesis by suppressing the inflammatory reaction and promoting corneal wound healing.

Matrix metalloproteinases (MMPs), a family of zinc-dependent extracellular endoproteinases, are one of the primary angiogenic factors in CNV. They have been demonstrated as essential factors in the formation and development of CNV by degrading components of the extracellular matrix and facilitating migration of vascular endothelial cells to the lesion [37].The rodent lack of MMP-1, MMP-13 is thought to function as the role of MMP-1 in humans. MMP-1 is native to the cornea and it degrades types I, II, and III collagens [38]. MMP-2 and MMP-9, also known as gelatin A and gelatin B, play crucial roles in angiogenesis [39]–[41].They degrade type IV collagen, which forms the filamentous structure and basement membranes. Previous studies have shown that minocycline could inhibit the activity of MMP- 2, -3, -8, -9, -12, and -13 [42]–[44]. In our study, the mRNA and protein levels of MMP-2, and MMP-9 and mRNA levels of MMP-13 were significantly reduced after administration of minocycline (60 mg/kg b.i.d. i.p.). This may partly contribute to the antiangiogenic function of minocycline on CNV by inhibiting the remodeling of burned corneal stroma. As for MMP-8, it is secreted by polymorphonuclear neutrophils (PMNs). Gabler et al. reported tetracycline could suppress neutrophil-mediated tissue damage by inhibiting their migration and by suppressing synthesis of oxygen radicals functions [45]. Doxycycline was also reported to reduce the activity of PMNs in patients with acute myocardial infarction [46].In this study, we did not find difference among the three groups in the mRNA expression of MMP-8 (neutrophil collagenase). Nonetheless, the number of infiltrated PMNs in the corneal stroma on day 14 was lower in the high-dosage group than in the control group. This may be due to the amount of PMNs, which might not have been enough to make a difference between the mRNA and its protein expression of MMP-8 on day 14 after burns. The infiltration of PMNs happens at as early as 6 hours, reaches a peak at 24–48 hours after injury, and then begins to decrease in number, due to apoptosis [1]. So, we need further investigation of the effect of minocycline on PMNs in the burned cornea at different checkpoints.

Minocycline has a long history of safe usage in clinics. It is recommended to be taken orally for acne vulgaris and for some sexually transmitted diseases at a dosage of 200 mg/d, and for neuroprotection in amyotrophic lateral sclerosis at a maximum dosage of up to 400 mg/day in humans [18]. Our results showed that minocycline effectively inhibited mice CNV at a dosage of 60 mg/kg twice a day by intraperitoneal injection. This dosage may be not safe for humans, but it is tolerable for mice. The median lethal dose (LD50) of minocycline in mice through intraperitoneal injection is about 310 mg/kg [47]. In a recent report, minocycline was even injected intraperitoneally at a first dose of 90 mg/kg and then twice 45 mg/kg in the next 9 hours for preventing traumatic brain injury in mice [48]. During our study, severe adverse side effects, including digestive disorder and even death directly related to the use of minocycline were not observed in the mice. So the dosage of minocycline (30 mg/kg and 60 mg/kg b.i.d i.p) was acceptable for mice research in this study. Otherwise, we did not investigate its pharmacokinetics, pharmacodynamics, safety and tolerability of systemic minocycline for ophthalmic use. For eye diseases, it may be better to make minocycline eye drops of effective concentration for topical medication, in order to avoid exposing the human body to the toxicity of high-dosage minocycline. However, there is no commercially available ophthalmic preparation of minocycline because of its unstable character under light exposure. Recently, Kaiser et al. has made minocycline nanoliposome-encapsulated for treating diabetic retinopathy in rats by subconjunctival injection [49]. In the future, we need determine the concentration of minocycline in the cornea for effectively inhibiting CNV and make minocycline with an appropriate delivery system, like eyedrops or ointment for topical application.

In summary, minocycline has more functions besides its antibiotic character, as shown in this study and in other reports. Minocycline may someday play a promising role in preventing CNV.
